# Prediction of Left Ventricular Remodeling after a Myocardial Infarction: Role of Myocardial Deformation: A Systematic Review and Meta-Analysis

**DOI:** 10.1371/journal.pone.0168349

**Published:** 2016-12-30

**Authors:** Olivier Huttin, Stefano Coiro, Christine Selton-Suty, Yves Juillière, Erwan Donal, Julien Magne, Nicolas Sadoul, Faiez Zannad, Patrick Rossignol, Nicolas Girerd

**Affiliations:** 1 Centre Hospitalier Universitaire de Nancy, Service de Cardiologie, Institut Lorrain du Cœur et des Vaisseaux, Nancy, France; 2 Centre d’Investigations Cliniques- Plurithématique 14–33, and Institut national de la santé et de la recherche médicale U1116, Nancy, France; 3 Centre Hospitalier Universitaire, Pôle de Cardiologie, Institut Lorrain du Cœur et des Vaisseaux, Vandoeuvre lès Nancy, Nancy, France; 4 Université de Lorraine, Nancy, France; 5 Département de Cardiologie & CIC-IT U 804, Centre, Hospitalier Universitaire de Rennes, LTSI, Université Rennes 1, INSERM 1099, Rennes, France; 6 Centre Hospitalier Universitaire Limoges, Hôpital Dupuytren, Department of Cardiology, Limoges, France; 7 Investigation Network Initiative Cardiovascular and Renal Clinical Trialists, Nancy, France; I2MC INSERM UMR U1048, FRANCE

## Abstract

**Aims:**

Left ventricular (LV) adverse or reverse remodeling after ST-segment elevation myocardial infarction (MI) is the best outcome to assess the benefit of revascularization. Speckle tracking echocardiography (STE) may accurately identify early deformation impairment, while also being predictive of LV remodeling during follow-up. This systematic analysis aimed to provide a comprehensive review of current findings on STE as a predictor of LV remodeling after MI.

**Methods:**

PubMed databases were searched through December 2014 to identify studies in adults targeting the association between LV remodeling and STE. Meta-regression was performed for longitudinal analysis.

**Results:**

A total of 23 prospective studies (3066 patients) were found eligible. Eleven studies reported an association between STE and adverse remodeling and twelve studies with reverse remodeling. Using peak systolic longitudinal strain, the most accurate cut-off to predict adverse remodeling and reverse remodeling ranged from -12.8% to -10.2% and from -13.7% to -9.5%, respectively. In smaller studies, assessment of circumferential strain and torsion showed additive value in predicting remodeling. Meta-regression analysis revealed that longitudinal STE was associated with adverse remodeling (pooled univariable OR = 1.27, 1.17–1.38, p<0.001; pooled multivariable OR = 1.38, 1.13–1.70, p = 0.002) while pooled ORs of longitudinal STE only tended to predict reverse remodeling (pooled OR = 0.75, 0.54–1.06, p = 0.09).

**Conclusions:**

This systematic review suggests that STE is associated with changes in LV volume or function regardless of underlying mechanisms and deformation direction. Meta-regression demonstrates a strong association between peak longitudinal systolic strain and adverse remodeling. Added STE predictive value over other clinical, biological and imaging variables remains to be proven.

## Introduction

Changes in left ventricular (LV) geometry after ST-elevation acute myocardial infarction (STEMI) is a complex phenomenon characterized by various phases. Remodeling is defined as the changes in LV end-diastolic volume (LVEDV) and/or end-systolic volume (LVESV) between discharge and late follow-up measurements [1,2]. Adverse remodeling is defined as a clinically significant increase in LVEDV whereas reverse remodeling is defined as a clinically significant decrease in LVESV, wall motion score index (WMSI) or LV ejection fraction (LVEF) improvement.

Although LVEF has traditionally been used as a predictor of major adverse cardiac events, its use as a sole predictor is currently under debate [3]. From a pathophysiological standpoint, adverse remodeling may lead to heart failure and ventricular arrhythmias, thus increasing the mortality rate [4]. Identification of patients with a high likelihood of either adverse or reverse LV remodeling after STEMI has therefore critical implications for risk stratification in the acute phase [1,5].

Depending on the severity of ischemic injury, deformation parameters derived from speckle tracking echocardiography (STE) have been introduced as new noninvasive echocardiographic parameters to quantitatively assess global and regional myocardial function [6]. The ability of STE-derived parameters to predict the extent and transmurality of myocardial necrosis after STEMI suggests their possible role in predicting subsequent LV remodeling [7]. Several studies have thus aimed to determine the predictive value of the various two and three-dimensional strain components for LV remodeling and clinical outcomes in patients with STEMI. However, the literature exploring the usefulness of STE in the prediction of LV remodeling has involved relatively small series.

Using a systematic review of the current literature, the present analysis aimed to assess the prognostic value of STE to predict LV adverse or reverse remodeling after a STEMI, based on published evidence.

## Materials and Methods

### Search strategy and study selection

This review adheres to the standards described in the Preferred Reporting Items for Systematic reviews and Meta-Analysis (PRISMA) statement [8]. An article was deemed relevant if it was a cohort study or a clinical trial of patients admitted with acute STEMI and if it reported STE measurement and LV remodeling data.

**Fig 1:**
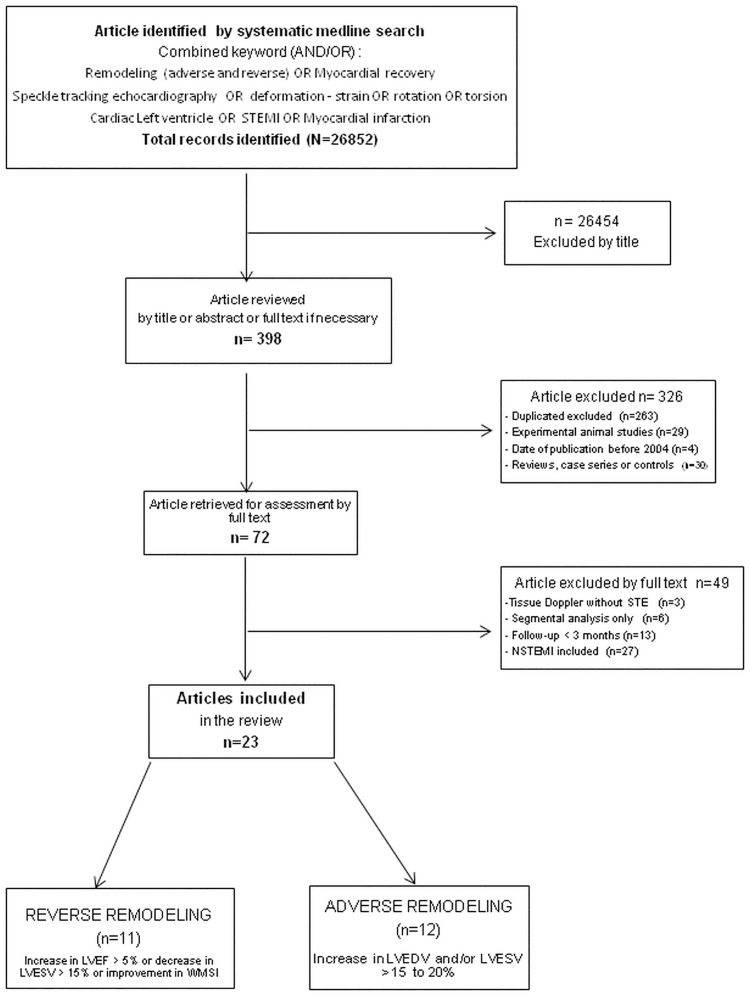
Search Flow Diagram. Literature Search Flow Diagram of the detailed process of selection of articles for inclusion in the current review by two independent experimented cardiologists.

A computerized Medline search of published articles was conducted from June 2004 (when deformation measurement by STE was validated in normal human LV as a novel index of systolic function [6,9]) through December 2014. Combined keywords (AND/OR) were searched: remodeling (reverse and adverse); myocardial recovery; speckle tracking echocardiography; deformation; strain; rotation; torsion; cardiac; left ventricle; STEMI; myocardial infarction. Reference lists of all relevant articles were searched manually for additional articles (Fig 1).

Focus was placed on patients with acute phase STEMI (two studies with mixed STEMI and NSTEMI population) and revascularization therapy with data on deformation imaging (using STE) at baseline (in the first week) and evaluation of changes in LV volume and ejection fraction during follow-up.

Peer-reviewed studies assessing the role of STE (two-dimensional and/or tridimensional (2D/3D) were included regardless of the analyzed direction of myocardial motion (longitudinal, circumferential or radial) and of the type of measured parameter (strain, strain rate, torsion or twist). Studies were selected if they reported correlation or diagnostic value of deformation imaging in predicting cardiac remodeling. Studies assessing myocardial deformation using tissue Doppler imaging were excluded from the analysis.

Studies were divided into two groups according to the reported type of LV remodeling:

- Adverse remodeling with an increase in LVEDV and/or LVESV by 15 to 20% [1.2];
- Reverse remodeling with myocardial recovery: improvement in wall motion score index or increase in LVEF greater than 5% or decrease in LVESV greater than 15% [3]. (Fig 1; Tables A-D in S1 File).

The association between STE before discharge and the degree of LV remodeling was evaluated, as assessed by a cut-off value or use as a continuous variable. The presence or absence of LV remodeling, as assessed using follow-up imaging tests, was considered as endpoint. The degree of association was also reported according to the available statistical analysis for each publication (Tables A-D in S1 File).

The reviewers (OH and SC) independently screened the titles and abstracts of identified reports. The full published reports of all abstracts selected by the reviewers were retrieved, and a second-step selection based on the inclusion criteria was performed. Reviewers were blinded to details of authorship or study results. Language (English) and age (adults) restrictions were imposed. Studies with less than a 3-month follow-up to assess LV remodeling as well as studies from the same center, studying the same population during the same time period, were excluded. Studies using tissue Doppler to assess strain or strain rate were excluded (Fig 1; Tables A and B in S1 File).

Specificity of each study (i.e., clinical outcomes or use of other imaging strategy) was noted. Duration of follow-up when given, characteristics of the study population, parameters studied, inter-group comparison and outcome along with statistical information (data for hazard ratio, relative risk, incidence and statistical significance) were collected. Reproducibility of global peak longitudinal strain (GLS) was also documented if available. A relationship between deformation and LV remodeling was defined as: i) positive if a variation in deformation parameters was significantly associated with LV remodeling or ii) negative if STE was not significantly associated with LV remodeling. The deformation parameter was considered as an absolute value of the number for all references indicating correlation between remodeling or LV function and strain. Hence, increases in GLS signify that the numerical value is becoming increasingly negative, and decreases are observed when LV function deteriorates and GLS becomes less negative. Circumferential strain is expressed as negative value, while radial strain, rotation and twist are expressed as positive values. The degree of association was also reported according to the available statistical analysis for each publication:

- Analysis based on receiver-operating characteristic (ROC) curves: the ability of continuous variables to predict remodeling was verified on the basis of ROC curve analysis. Overall accuracy, sensitivity, specificity, and positive and negative predictive values for optimal cut-off points were calculated.
- Univariate association (odds ratio (OR) or B coefficient) for the specific STE component. Importantly, the associations were reported for a 1% increase in strain values without transformation (for instance, for an increase from -16% to -15%, which represents a worsening of strain).
- Independent predictors of LV remodeling were defined using multivariate analysis (using various variable selection strategies).

Other important and potentially confounding baseline clinical and echocardiographic predictors known to influence outcome and LV systolic function after STEMI were similarly tested for their ability to predict change in LVEDV over time. These parameters were usually included as a candidate variables in the multivariable analysis to predict remodeling (most frequent were: location (anterior), multivessel disease, time reperfusion, Killip class, CPK, troponin, WMSI and LVEF). Candidate variables in the multivariable analysis or/and other significant variables are specified in supporting information (Tables C and D in S1 File).

When available in the original publications, the net reclassification improvement index (NRI), C index or Chi-2 value associated with the adjunction of strain variables in basal models are provided. The supporting PRISMA checklist is available as supporting information (see S2 File).

### Meta-analysis methods

Meta-regression analysis of 9 studies studying Longitudinal STE was performed. The pooled ORs were weighted by the inverse of their variance and combined according to a fixed-effects model in the absence of heterogeneity and according to a random-effects model [10] in the presence of heterogeneity, as previously used by our group [11]. Cochran’s Q and I2 tests were used to analyze heterogeneity, with the latter considered present if the p-value of the Q test was <0.10 and if I2>50% [12]. Publication bias was examined by conducting a funnel plot by the Egger regression model [13]. Funnel plot asymmetry was tested by drawing a regression line. A P value inferior to 0.1 for the slope coefficient was considered to represent significant asymmetry reflecting publication bias.

## Results

### Characteristics of the included studies

The search yielded an analysis of 23 studies, including 3066 patients. There were 11 studies assessing adverse remodeling [14–24], 11 assessing reverse remodeling [25–35] and 1 assessing both outcomes [36]. All patients reported in these studies had a successfully revascularized first STEMI (as defined by TIMI flow>2) except for 2 studies in which both STEMI and NTEMI patients were included [19,36]; only 2 studies included medical revascularization with thrombolysis. Most studies included a mixed STEMI population, with both anterior and inferior localizations; only 5 studies [15, 20, 26, 28, 32] included patients with anterior STEMI. All patients underwent 2D or 3D deformation imaging in the first week following the acute event, with most patients undergoing the echocardiographic assessment within 48-72h of their hospital admission (Tables A and B in S1 File). Sixteen of 23 studies had a dedicated analysis of reproducibility with a correlation coefficient of variation.

### Deformation and remodeling

The majority of the studies identified strain to be independently associated with LV remodeling.

#### Longitudinal strain STE

The use of GLS most consistently detected the change in LV volume during follow-up among the different studies [14–16, 24, 25, 30–34, 36,37]. The determinant cut-off value of GLS for adverse remodeling ranged from -12.8% to -10.2% and from -13.7% to -9.5% for reverse modeling. In multivariate adjusted analysis, GLS provided significant incremental value over clinical and conventional echocardiographic variables in predicting global LV function improvement (C-statistic index) [29] or LV remodeling [16–18].

#### Circumferential and radial strain STE

In small studies, evaluation of circumferential deformation showed an additive value of Peak Circumferential Strain (PCS) and Peak Radial Strain (PRS) in predicting remodeling. PCS provided a stronger association with similar prediction for adverse remodeling [18, 20, 27, 35]. In the VALIANT Echo study, Hung et al. clearly demonstrated that PCS but not GLS was predictive of LV remodeling after adjustment for clinical and echocardiographic variables at 20 months [18]. Bonios et al. also demonstrated the appealing value of PCS in apical segments in predicting adverse remodeling [20].

#### Rotation, torsion and twist

Counter-directional rotation results in a wringing motion of the heart, known as LV torsion (LVT) or twist. LV twist is the net rotational difference between the apex and base of the LV expressed in degrees. A total of 5 different studies assessing the phenomenon of base-to-apex gradient in rotation and LV remodeling were found [20–23, 26, 28]. Jang et al., Nucifora et al. and Park et al. provided predictive values of rotational characteristics and peak LV torsion which demonstrated modest, albeit significant incremental value over clinical echocardiographic variables in detecting LV remodeling in acute phase of an ischemic event (Chi-square values) [21, 22, 28].

### Meta-regression

Meta-regression was only performed for 2D GLS in the present series since the number of studies investigating other echo parameters was too limited to perform a statistically valuable analysis.

#### Meta-regression of univariable OR with 2D GLS as explanatory variable and adverse remodeling as outcome

**Fig 2:**
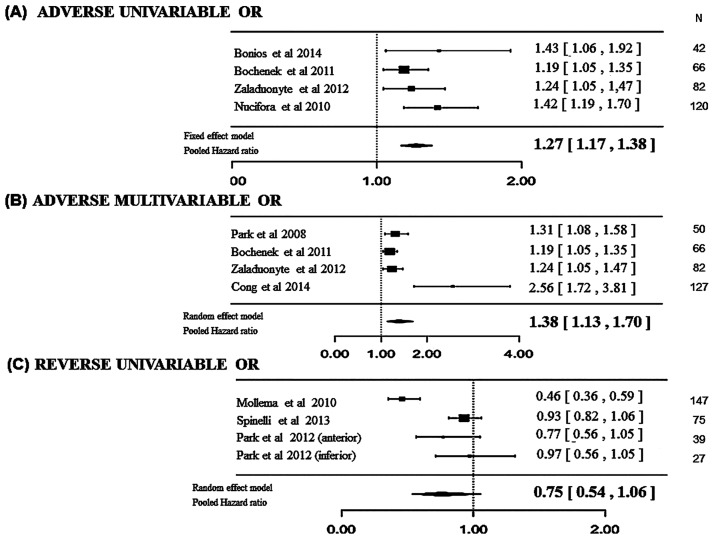
Forest Plot. Pooled Odds Ratios (OR) for the association of global left ventricular peak systolic longitudinal (GLS) strain evaluated in acute phase of STEMI and left ventricular remodeling at follow-up. (A) Univariable OR with 2D GLS as explanatory variable and adverse remodeling as outcome were available in 4 studies (pooled OR = 1.27, 1.17 to 1.38, p<0.001) (B). In addition, multivariable OR were available in 4 studies (pooled OR = 1.38, 1.13–1.70, p = 0.002). (C) Univariable OR with 2D GLS as explanatory variable and reverse remodeling as outcome were available in 3 studies (pooled OR = 0.75, 0.54–1.06, p = 0.09).

Univariable OR with 2D GLS as explanatory variable and adverse remodeling as outcome were available in 4 studies [16, 17, 20, 22]. The meta-regression showed that the odds of adverse remodeling increased by more than 20% for each 1% increase in relative 2D GLS value (i.e. a 1% decrease in absolute value of 2D GLS) (pooled OR = 1.27, 1.17 to 1.38, p<0.001, in a fixed-effects model) (Fig 2).

#### Meta-regression of multivariable OR with 2D GLS as explanatory variable and adverse remodeling as outcome

In addition, multivariable ORs were available in 4 studies, 2 of which stemmed from the same studies with available univariable OR [15–17, 38]. To account for this heterogeneity, we constructed a random-effects model, which provided a very similar association (pooled OR = 1.38, 1.13–1.70, p = 0.002) as that reported for the univariable OR (Fig 2).

#### Meta-regression of multivariable OR with 2D GLS as explanatory variable and reverse remodeling as outcome

Univariable OR with 2D GLS as explanatory variable and reverse remodeling as outcome were available in 3 studies [25, 26, 28], providing 4 ORs (Park et al. reported 2 ORs, one for anterior MI and one for inferior MI). In a random-effects model, a 25% decrease in the odds of reverse remodeling was observed for each 1% increase in 2D GLS value, although not significant (pooled OR = 0.75, 0.54–1.06, p = 0.09). Fig 2 shows the impact of GLS on adverse and reverse LV remodeling.

## Discussion

Several key messages emerge from this systematic review and meta-analysis: 1) overall, an alteration in STE-derived measurements of myocardial deformation appears to be an early and powerful predictor of adverse LV remodeling after STEMI; 2) 2D GLS was the most studied variable and was significantly associated with adverse LV remodeling in meta-regression analyses; 3) additional evidence is required to determine whether 2D GLS is a significant predictor of reverse remodeling (only a trend could be identified in the present meta-regression) and whether other less commonly used components of deformation are significant predictors of LV remodeling; 4) using a variety of methods, several studies demonstrated that STE-derived strain variables allowed to significantly increase the accuracy of remodeling prediction on top of conventional clinical and imaging criteria.

### Strategy of left ventricular remodeling assessment after a STEMI

Despite the high success rate of current revascularization by percutaneous coronary interventions (PCI), adverse LV remodeling nonetheless occurs in one third of patients following acute MI (12 to 44%) with a rate of LV recovery ranging from 12 to 54%. Some of the variability in the prevalence of remodeling between studies may be due to differences in study populations, revascularization mode and timing of imaging. The changes in LV volume during follow-up is a prerequisite for estimating the degree of LV remodeling after a STEMI although different times, methods and threshold are currently used. Adverse remodeling is usually defined by an increase in LVE(S)DV of 15 to 20% from baseline [1–3]. The assessment of LV functional recovery is inconsistently defined by an increase in LVEF (> 5 to 10%), a decrease in LVESV (>15%) or an increase in wall motion score index [39]. The first assessment is usually performed at discharge by Simpson biplane 2D LVEF on transthoracic echocardiography (TTE). In several studies, the first TTE was performed within the first 24h [24, 28, 34], most of the time within the first 48h and never after day 10. This differential timing of assessment may lead to an over- or under-estimation of remodeling due to rapid dynamic changes occurring during the first week. However, estimates of LV volumes derived from 2D images are subject to variability imposed by selection of the imaging plane and inaccuracies in LVESV analysis. Cardiac magnetic resonance (CMR) measurements are now considered the standard for measurement of volumetric parameters given their high reproducibility in detecting small changes between two time points. Only two studies included CMR evaluation at baseline while none assessed LV volume change by CMR at follow-up [33, 35].

Nevertheless, 2D TTE remains the predominant clinically applicable method of choice based on broader availability. It is the primary reason why standard LV volume measurements need to be complemented by deformation information in order to strengthen the potential of TTE to assess infarct size and subsequently to predict LV remodeling at no additional cost and very limited additional time [40].

### What is the best STE parameter for predicting left ventricular remodeling?

**Fig 3:**
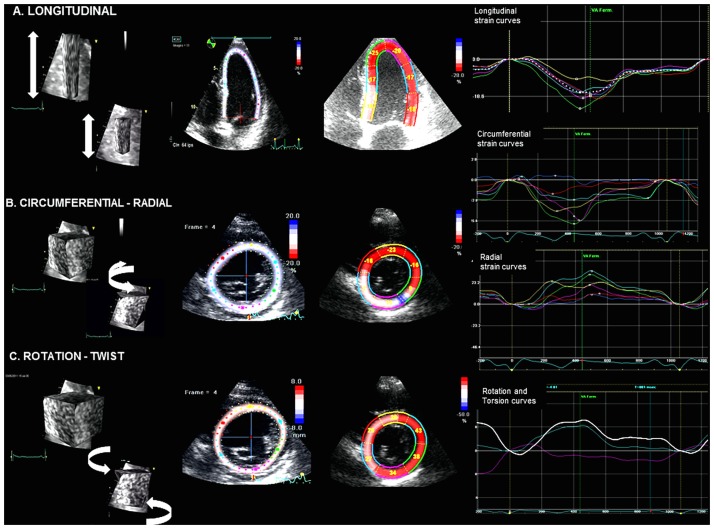
Speckle Tracking Echocardiography (Illustrative Figure). Patient with first inferior STEMI in acute phase STE with peak systolic strain measurements by GE Vivid EchoPAC software: (A) apical 4 chamber view with longitudinal strain calculation, (B) parasternal short axis view with circumferential (CS), radial strain (RS), and (C) LV twist calculation based on rotation values. The left panels show the direction (arrows) in which the various strain parameters are being measured. The middle panels demonstrate the region of interest and segmental strain values (except for circumferential strain) while the right panels illustrate the regional strain curves.

STE-derived parameters are markers of LV function which more closely reflect intrinsic and subclinical myocardial impairment than traditional parameters by assessing the active component of myocardial deformation. Strain systolic values are lower in the infarct territory than in normal myocardium and are related to the degree of infarct transmurality extent [7] (Fig 3). In the adjacent myocardium near the infarcted zone, abnormal deformation due to stunning, transient ischemia and local loading conditions is generally observed and plays an important role in LV remodeling [41]. For all of these reasons, strain parameters would appear to be more efficient than LVEF alone in evaluating the potential of LV remodeling after an acute event.

Numerous studies have shown the potential diagnostic value of 2D PCS in discriminating nontransmural from transmural infarction [42]. The extent of transmural infarction with significant impairment of LV torsion represents the first step of a vicious cycle of progressive LV dilatation and LV function non-recovery. Middle and sub-epicardial layers further contribute to thickening and to radial and circumferential function. These fibers are a key determinant of preserved global LV function and serve as a short-axis restraint to prevent further LV geometric expansion [18]. However, 2D assessment of torsion and PCS requires acquisition of 3 short axis views with potential changes in heart rate and loading conditions between the various acquisitions with a resulting influence on deformation values. The main limitation is the quality of the apical and basal parasternal views and the tracking accuracy which, at times, can be difficult to obtain in patients in the acute phase of a cardiac event. Due to the scarcity of available data in the literature, it was not possible to perform a meta-regression in this setting. Additional studies with particular focus on these parameters are therefore indicated.

Sub-endocardial fibers generate the most significant contribution to longitudinal function and are attenuated independently of scar transmurality. In addition to demonstrating better correlation with infarct size than LVEF, GLS also allows prognostic evaluation with a good clinical value for predicting heart failure [43] and could logically be considered as a marker of remodeling with the ability to predict subsequent LV dysfunction. The strength of GLS is its reproducibility and accuracy due to the semi-automated nature of the measurements. This technique is easy and quick to perform in clinical routine, obtained from the 3 apical views regardless of the clinical status of the patient or the ability to stop breathing or to turn on the left side. Since 2D GLS is the most commonly used strain variable, we were able to perform both a univariable odds ratio and a multivariable odds ratio meta-regression. These analyses revealed that for each 1% decrease in 2D GLS (a 1% GLS decrease in absolute value of 2D GLS), the odds were increased by 27% to 38%. This demonstrates the strong association between GLS and adverse remodeling observed in the available literature. Meanwhile, the meta-regression analysis of associations with reverse remodeling provided only a trend (p = 0.09), thus suggesting that additional studies are warranted in order to draw definitive conclusions in this regard.

Alternatively, a 3D deformation analysis in acute phase of MI may most likely prove more efficient since it takes into account both radial and circumferential deformation in the same acquisition.

### Perspectives: Added value on top of clinical and biological parameters

LV volume changes and prediction of cardiac remodeling are not clearly defined and often challenging. Nevertheless, global and segmental LV remodeling in post MI has a strong impact on medical decisions in ischemic heart disease. Usual conventional clinical and echocardiographic variables can predict LV remodeling during follow-up, namely anterior wall infarction, time to reperfusion, marker of myocardial injury, diabetes mellitus and worse segmental LV contractility (higher wall motion score index) [44]. Several imaging techniques, such as dobutamine stress and myocardial contrast echocardiography, have been applied for this purpose; however, they do not fulfill the unmet clinical need due to their difficulty of implementation [22, 28].

Given that several variables are already known to predict remodeling, the usefulness of strain variables should be assessed on top of these variables. Nevertheless, only one of these studies, in a recently published article by Joyce et al., identified a additive prognostic value over clinical, biological and classical echocardiographic factors (including left anterior descending artery and WMSI)[14]. However, baseline LV volumes and BNP levels (known predictors of remodeling) were not taken into account. Consequently, further studies are needed to fully assess the gain in information provided by STE. By nature, only large-scale studies similar in size to that performed by Joyce et al., or studies providing a more accurate assessment of LV volume changes by means of CMR would be able to assess the predictive value of strain variables on top of usual variables [14]. Ultimately, an approach using strain as the primary marker of remodeling to initiate cardioprotective therapy needs could be compared with a traditional TTE-based and biomarker approach [45].

### Study limitations

A main limitation of the present analysis is that most studies were single-center series with small sample sizes. In addition, various imaging techniques and timing of imaging following MI were used, leading to significant heterogeneity of results. There is currently no large study cohort comparing the various strain components. Measurements of STE parameters were performed offline using dedicated software and after acquisition by a GE Vivid 7 or 9 in most instances (19/23). A few studies did assess strain components using other systems, including Siemens (1 study), Philips (2 studies) and Toshiba (1 study) systems. Recurrent myocardial ischemia, loading condition and therapy may change cardiac volume and thus influence strain values between the first hours after revascularization and discharge. Another limitation is that only a major database (Medline) was used for search and study selection.

One important limitation of our meta-analysis is the absence of available individual data. Importantly, the studies included in our meta-regression used different cut-offs for adverse remodeling (an increase in LVEDV or /and LVESV by 15 to 20%) and reverse remodeling (improvement in WMSI or increase in LVEF greater than 5% or decrease in LVESV greater than 15%). In addition, the studies included in this meta-analysis used various 2DGLS cut-offs. However, cut-offs values of 2DGLS ranged from -12.5 and 10.2, and most studies found an optimal cut-off very close to -11, we would promote -11 as the most evidence-based cut-off so far. However, further larger studies, namely multicenter studies with derivation and validation cohorts, should validate the most appropriate cut-off for both adverse and reverse remodeling after MI as well as the 2DGLS cut-off for both these outcomes. In the meanwhile, even if it does not provide definitive evidence regarding cut offs, our meta-analysis provides evidence for the significance of the association between strain and remodeling after MI.

## Conclusions

In this pooled analysis, longitudinal strain appears to be the more consistent and widely available approach to accurately predict changes in LV volume late after MI, reinforcing the value of STE-derived myocardial deformation parameters as early predictors of LV remodeling after a STEMI. Non-invasive measurement of cardiac mechanics is reproducible and inexpensive, allowing to select a subset of STEMI patients who may require closer follow-up and more aggressive management. An important limitation of this technique is underscored by radial and circumferential strain, both affected by a low reproducibility and with limited feasibility in clinical practice; additionally, despite a large number of studies, the current literature is constrained by the preponderance of non-uniform single-center studies. However, a more comprehensive multi-parametric approach to assess LV remodeling, including deformation imaging in combination with new biomarkers and CMR, is likely to confirm the present meta-regression results.

## Supporting Information

S1 File **Table A in S1 File. Adverse remodeling studies.** Summary of studies that have used advanced myocardial mechanics parameters to demonstrate adverse remodeling in patients with first myocardial infarction **Table B in S1 File. Reverse remodeling studies.** Summary of studies that have used advanced myocardial mechanics parameters to demonstrate reverse remodeling in patients with first myocardial infarction **Table C in S1 File. Speckle tracking echocardiography and adverse remodeling.** Association of deformation values with cardiac adverse left ventricular remodeling **Table D in S1 File. Speckle tracking echocardiography and reverse remodeling.** Association of deformation values with cardiac reverse left ventricular remodeling.
(DOCX)

S2 File PRISMA Checklist. (DOCX)
