# Supplementary material for: Prediction of Left Ventricular Remodeling after a Myocardial Infarction: Role of Myocardial Deformation: A Systematic Review and Meta-Analysis
Source: PLoS One. 2016 Dec 30;11(12):e0168349. doi: 10.1371/journal.pone.0168349 (PMC5201304; doi:10.1371/journal.pone.0168349)
Supplement: S1 File — Table A in S1 File. Adverse remodeling studies. Summary of studies that have used advanced myocardial mechanics parameters to demonstrate adverse remodeling in patients with first myocardial infarction Table B in S1 File. Reverse remodeling studies. Summary of studies that have used advanced myocardial mechanics parameters to demonstrate reverse remodeling in patients with first myocardial infarction Table C in S1 File. Speckle tracking echocardiography and adverse remodeling. Association of deformation values with cardiac adverse left ventricular remodeling Table D in S1 File. Speckle tracking echocardiography and reverse remodeling. Association of deformation values with cardiac reverse left ventricular remodeling. (DOCX) [file pone.0168349.s001.docx]

**Table A :** Summary of studies that have used advanced myocardial mechanics parameters to demonstrate adverse remodeling in patients with first myocardial infarction

|  | **First author**  **(Ref. #), Year** | **n** | **Main STE PARAMETER** | **STUDY DESIGN** | **FEATURES** | | | | **METHOD FOR**  **ASSESSMENT OF**  **LV REMODELING** | **ECHO-MACHINE**  **VENDOR** | **TIME OF FIRST**  **ASSESSMENT** | **TIMING OF REMODELELING** | **REPRODUCBILITY**  **Agreement (%) or ICC** | **PERCENTAGE REMODELING**  **N (%)** | **PARTICULAR FEATURES** |
| --- | --- | --- | --- | --- | --- | --- | --- | --- | --- | --- | --- | --- | --- | --- | --- |
|  |  |  |  |  | **AGE** | **LVEF** | **PTCA (%)** | **LOCATION** ANT/ N ANT % |  |  |  |  |  |  |  |
| **ADVERSE REMODELING** | Joyce et al. [1], 2014 | 1041 | **2D PLS** | Retro | 60±12 | 47±9 | 100% | 46% | Echo | GE Vivid 7 | <48h | 3 - 6 Mo | Inter  0.1± 2.2  Intra  0.2 ± 0.8 | Use as a continuous variable | - |
|  | Park et al. [2] 2008 ^†^ | 50 | **2D PLS** | Pro | 56±13 | 47±11 | 88% | 100% | Echo (LVEDV >15%) | GE Vivid 7  Echopac PC 6.0.0 | <48h | 15±8 Mo | Inter  0.78  Intra  0.94 | 22 (44%) | Clinical events  (Survival, HF, Hospitalization) |
|  | Bochenek et al. [3] 2011 ^*†^ | 66 | **2D PLS** | Pro | 60±10 | 50±9 | 100% | 47% | Echo (LVEDV >20%) | GE Vivid 7 Echopac PC 6.0.0 | 4–6 d | 3 Mo | Intra  1.38%  Intra  1.81% | 22 (33.3%) | 2D / 3D TTE  Segmental analysis |
|  | Atiok et al. [4] 2014 | 93 | **2DPLS** | Pro | 60±11 | 56±10 | 100% | 46% | Echo (LVESV >15%) | GE Vivid 7 Echopac PC 0.5.2 | < 48h | 6 Mo | Intra  7.9%  inter  9.1%. | 11 (12%) | 59% (55/92) of ST+  37 NSTEMI  CMR |
|  | Zaladuonyte et al. [5] 2011 ^*†^ | 82 | **2D PLS**  **2D PCS** | Pro | 58±9 | 53±10 | 100% | 56% | Echo (LVEDV >15%) | GE Vivid 7  Echopac PC 6.1 | 24-72h | 4 Mo | Inter  0.95  Intra  0.96 | 28 (34%) | 4 - 2 chamber |
|  | Hung et al. [6] 2010 | 311 | **2D PLS**  **2D PCS** | Pro | 63±12 | 39±10 | 21% | 77% | Echo (LVESV >15%) | Siemens | 5+-2.5d | 20 Mo | Good  (no values reported) | NA | Clinical outcomes (all cause and CV deaths, HF) |
|  | Haberka et al [7] 2014 | 63 | **2D PLS**  **Torsion** | Retro | 62±12 | 57±8 | 100% | 18% | Echo (LVEDV and /or LVESV >20%) | Toshiba | 72h | 3 Mo | no | 17 (27%) | 4 and 2C  23 NSTEMI  Clinical outcomes |
|  | Bonios et al. [8] 2014 ^*^ | 42 | **2D PLS**  **2D PCS**  **Torsion** | Pro | 57±14 | 47±7 | 100% | 100% | Echo (LVESV>15%) | GE Vivid 7  Echopac PC 7.0 | 96h | 3 Mo | No for 2DPLS | 13 (31%) | - |
|  | Jang et al. [9] 2010 | 91 | **Torsion** | Pro | 64±11 | 52±-17 | 100% | 51% | Echo (LVEDV >20%) | GE Vivid 7 | 72h | 6 Mo | Intra  0.94  Inter  0.91 | 23 (25%) | - |
|  | Nucifora [10] 2010 ^*^ | 120 | **2D PLS**  **Torsion**  **Twist** | Pro | 59±10 | 48±9 | 100% | 46% | Echo (LVESV>15%) | GE Vivid 7  Echopac PC 7.0 | 48h | 6 Mo | No for 2DPLS only for twist | 19 (16%) | Myocardial contrast TTE |
|  | Abate et al. [11] 2014 | 213 | **2D PLS**  **3D PLS Twist** | Pro | 61 (52-69) | 49 (44-53) | 100% | 42% | 3D echo (LVESV>15%) | Philips I33  Qlab 7.0 | <48h | 6 Mo | No for 2DPLS only for twist | 44 (21%) | - |
|  | Cong et al. [12] 2014 ^†^ | 127 | **2D PLS** | Pro | 60+/-12 | 52+/-5 | 100% | 41% | Echo (LVESV>15%) | GE Vivid 7  Echopac PC 6.0 | <24h | 6-9Mo | no | 41 (32%) | Late PTCA (>6h)  Clinical outcomes |
| *LV: left ventricle; EF ejection fraction; ESV: end systolic volume; EDV: end diastolic volume WMSI: wall motion score index, ANT: Anterior; CMR: cardiac magnetic resonance; CKMB: creatine phosphokinase MB; Trop: troponin T; Mo: Months, y: years, d: days. GE: General Electric; CV: cardiovascular; HF: Heart failure; STE: Speckle tracking echocardiography; 2D/3D PLS: Two/Three dimensional peak longitudinal strain; 2DPCS: 2D peak circumferential strain; 2DPRS: 2D peak radial strain; 2Dprin: 2D principal strain; Ap: apical; Bas: Basal; Rot: Rotation; subepi: subepicardial; subend: subendocardial; PTS: Peak transverse strain; FU: follow-up; STEMI: ST elevation myocardial infarction; NSTEMI: non ST* | | | | | | | | | | | | | | | |

**Table B :** Summary of studies that have used advanced myocardial mechanics parameters to demonstrate reverse remodeling in patients with first myocardial infarction

| **REVERSE REMODELING** | **First author**  **(Ref. #),Year** | **n** | **Main STE PARAMETER** | **STUDY DESIGN** | **FEATURES** | | | | **METHOD FOR**  **ASSESSMENT OF LV REMODELING** | **ECHO-MACHIN**  **VENDOR** | **TIME OF FIRST** | **TIMING OF**  **REMODELING** | **REPRODUCBILITY**  **Agreement (%) or ICC** | **PERCENTAGE REMODELING N (%)** | **PARTICULAR FEATURES** |
| --- | --- | --- | --- | --- | --- | --- | --- | --- | --- | --- | --- | --- | --- | --- | --- |
|  |  |  |  |  | **AGE**  **(yrs)** | **LVEF**  **(%)** | **PTCA**  **(%)** | **LOCATION ANT/ N ANT (%)** |  |  |  |  |  |  |  |
|  | Atiok et al. [4] 2014 | 93 | **2D PLS** | Pro | 60±11 | 56±10 | 100% | 46% | Echo (LVEF >5%) | GE Vivid 7 | < 48h | 6 Mo | Intra  7.9%  inter  9.1% | 11(12%) | 59% (55/93) of STEMI |
|  | Mollema et al. [13] 2010 ^*^ | 147 | **2DPLS** | Pro | 61±11 | 48±7 | 100 | 54¨% | Echo (LVEF >5%) | GE Vivid 7  Echopac PC 6.1 | < 48h | 1 yr | no | 77 (52%) | Resting myocardial SPECT imaging |
|  | Spinellli et al. [14] 2013 ^*^ | 75 | **2DPLS**  **Twist**  **Torsion** | Pro | 52±11 | NA | 100 | 100% | Echo (>10% ESV) | GE Vivid 7  Echopac PC 8.0.0 | 36- 48 h | 6 Mo | Intra  0.97  Inter  0.93 | 25 (33%) | TTE before and after PCI  Outcomes (2y fu) |
|  | Carasso et al. [15] 2013 | 20 | **2D PLS**  **2D PCS**  **2D PRS**  **2D prin S** | Pro | 54±9 | NA | 100 | 65% | Echo WMS improvement | Siemens  (velocity vector imaging or GE) | 3-5 days | 4 Mo | no | ND | Segmental analysis Radionuclide  MPI score |
|  | Park et al. [16] 2012 ^*^ | 66 | **2DPLS**  **Torsion**  **Bas torsion** | Pro | 58±15 | 50±7 | 100 | 53% | Echo (WMSI > 2 grade 2 contiguous segments) | GE Vivid 7  Echopac PC 10.0. | <24h | 6 Mo | No  Only for rotation | 39 (59%) | ANT and INF MI separate  Only positive for ant |
|  | Abate et al. [17] 2011 | 153 | **3D PLS** | Pro | 59±11 | 47±7 | 100 | 45% | Echo (LVEF >5%) | GE Vivid 7 - e9  EchopacPC 110.0. | < 48h | 6 Mo | Variability reported as < 4% | 67 (44%) | - |
|  | Szymczyk et al. [18] 2014 | 96 | **2D PLS**  **2D PTS** | Pro | 58+10 | 50±7 | 100 | 51% | Echo (ND) | GE Vivid 7  Echopac PC 6.1.0. | 7-12d | 12 Mo | no | ND | low-dose dobutamine stress TTE |
|  | Park et al. [19] 2010 | 20 | **2D PLS** | Pro | 59±13 | ND | 100 | 100% | Echo (WMS improved >1 grade 2) | GE Vivid 7  Echopac PC 10.0.0. | 3-5 d | 6 Mo | Inter  3.9%  Intra  2.2%, | 10 (50%) | -- |
|  | Shehata et al. [20] 2014 | 50 | **2DPLS** | Pro | 56±5 | 40±7 | 100 | 100% | Echo (LVEF >5%) | GE Vivid 7 | ND | 4 Mo | no | 24 (48%) | Dobutamine for viability |
|  | Bergerot et al. [21] 2014 | 69 | **2DPLS** | Pro | 59±13 | ND | 100 | 58% | Echo (ND) | GE Vivid 7 | >3 d | 6 Mo | Inter  11.8%  Intra  2.6% | ND | CMR for infarct size and MVO |
|  | Korosoglou et al. [22] 2008 | 36 | **2DPLS** | Pro | 63±9 | NA | 100 | 72% | Echo (LVEF >10%) | Philips  (Qlab) | 12±9 h | 4-6 Mo | no | 19 (53%) | Perfusion (myocardial contrast echocardiography) |
|  | Orii et al. [23] 2014 | 35 | **2D PLS**  **2D PCS**  **2D PRS** | Pro | 66±11 | 48±4 | 100 | 59% | Echo (ND) | GE Vivid 7  Echopac PC | 8 d | 7±2Mo | inter-  0.91  Intra  0.83 | 19 (54%) | CMR and segmental analysis |
| *LV: left ventricle; EF ejection fraction; ESV: end systolic volume; EDV: end diastolic volume WMSI: wall motion score index, ANT: Anterior; CMR: cardiac magnetic resonance; CKMB: creatine phosphokinase MB; Trop: troponin T; Mo: Months, y: years, d: days. GE: General Electric; CV: cardiovascular; HF: Heart failure; STE: Speckle tracking echocardiography; 2D/3D PLS: Two/Three dimensional peak longitudinal strain; 2DPCS: 2D peak circumferential strain; 2DPRS: 2D peak radial strain; 2Dprin: 2D principal strain; Ap: apical; Bas: Basal; Rot: Rotation; subepi: subepicardial; subend: subendocardial; PTS: Peak transverse strain; FU: follow-up; STEMI: ST elevation myocardial infarction; NSTEMI: non ST* | | | | | | | | | | | | | | | |

**Table C :** Association of deformation values with cardiac adverse LV remodeling

|  | **First author**  **(Ref. #),**  **Year** | **STE PARAMETER** | **Association** | **AUROC**  **(CI 95%)** | **Determinant Cut-off value** | **Sensitivity, Specificity (%)** | | **UNIVARIATE ASSOCIATION**  **OR or B** | **MULTIVARIATE ASSOCIATION**  **OR or B** | **Candidate variables in the multivariable analysis** | **Other significant variables in the multivariable analysis** | **Predictive values**  **(C-Index- NRI or Khi2)** |
| --- | --- | --- | --- | --- | --- | --- | --- | --- | --- | --- | --- | --- |
| **ADVERSE REMODELING** | Joyce et al. [1] 2014 | **2DPLS** | + | ND | ND | ND | ND |  |  | age, sex, diabetes mellitus, Location , multivessel disease, Killip class, CPK troponin glucose, time, mitral regurgitation, discharge HR | Higher WMSI was associated with LVr, as were male sex, LAD, higher discharge, heart rate and troponin, and lower LAVI | LVGLS>15% was associated with a 6.7 (2.8-11) increase in LVEDV 3 months  And 10 (6.6-14) at 6 Mo  NRI on top of the other baseline characteristics=0.14(0.00-0.29) |
|  | Park et al. [2] 2008 ^†^ | **2DPLS** | + | 0.85 [0.73-0.97] | -10.2% | 91 | 86 |  | 1.31 [1.08-1.58]^†^ | WMSI, Time reperfusion, LVEF, CK-MB, E DT, E velocity, 2DPLS, DTI Strain | 2DSDTI | - |
|  | Bochenek et al. [3] 2011 ^*†^ | **2DPLS** | + | 0.77 | -12.5% | 69 | 79 | 1.2 [1.0-1.6] ^*^ | 1.19; [1.04–1.30]^†^ | ANT location, GLS, WMSI , leukocyte count, troponin, time to reperfusion, Max ST-segment elevation | diabetes mellitus | - |
|  | Atiok et al. [4] 2014 | **2DPLS** | + | 0.81 | -12.8% | 82 | 81 |  |  | - | - | - |
|  | Zaladuonyte et al. [5] 2012 ^*^ | **2DPLS** | + | 0.78 [0.67-0.89] | -11.6% | 78 | 73 | 1.21 [1.07-1.37]^*^ | 1.24 [1.05–1.48]^†^ | LAD, LVESV,WMSI, LVEF, leukocytes count value, PLS  early diastolic LS | LAD | - |
|  | Hung et al. [6] 2010 | **2DPLSR**  **2DPCSR** | + | - | - | - | - | - | - | - | Circumferential SRs but not longitudinal SRs was predictive of LVR | - |
|  |  |  |  |  |  |  |  |  | 1.3 [1.1 to 1.4] |  |  |  |
|  | Haberka et al. [7] 2014 | **2D PLS 2C** | + | - | - | - | - | - | - | - | Significant association for LVR as a covariate  (P = 0.015) | - |
|  |  | **2D PLS 4C** | - | - | - | - | - | - | - |  |  |  |
|  |  | **Torsion** | - | - | - | - | - | - | - |  |  |  |
|  | Bonios et al. [8] 2014 ^*^ | **2DPLS** |  |  |  |  |  | 1.43 [1.06-1.92]^*^ | - | apical CS, global LS, CPK max, and torsion | CPK | - |
|  |  | **2DCS Ap** | + | 0.98 | -11% | 100 | 96 | 2.56 [1.25-5.26] | + ND |  |  |  |
|  |  | **Torsion** |  |  |  |  |  | 0.37 [0.14-1.01] |  |  |  |  |
|  | Jang et al. [9]2010 | **Ap rot** | + | 0.76 | 5.7° | 87 | 52 | 0.57 [0.43-0.75] | ‡ | WMSI, LVEF Ap rotation, Torsion, DT, E’ velocity LVEDV, LVESV, LAD lesion | LVEF | - |
|  |  | **Bas rot** | - | - | - | - | - | 1.31[1.04-1.64] | ‡ |  |  |  |
|  |  | **Torsion** | + | 0.79 | 1.7°/cm | 84 | 78 | 0.12[0.04-0.37] | 0.16 [0.04-0.58] |  |  |  |
|  | Nucifora et al. [10] 2010^*^ | **2D PLS** | - | - | - | - | - | 1.43 [1.19-1.71] | ‡ | LAD, troponin T,  LVESV, LVEF, WMSI, diastolic dysfunction,  2DPLS, torsion, MPI. | MPI | - |
|  |  | **Torsion** | + | 0.92 | 1.44°/cm | 95 | 77 | 0.72 [0.62-0.82] | 0.77 [0.65-0.92] |  |  | X²=48.6 - C-stat 0.93 on top of clinical standard TTE and contrast echo |
|  | Abate et al. [11] 2014 | **Subendo twist** | + | 0.82 | 9.38° | 70 | 80 | 0.72 [0.64-0.81] | ‡ | subepi -subendo  twist, LVEDV, LVEF, troponin T | troponin T | X²=83.2 C-stat 0.91 |
|  |  | **Subepi-twist** | + | 0.96 | 5.17° | 95 | 86 | 0.21 [0.12-0.36] | 0.24 [0.13–0.43] |  |  | X ²=142.8 - C-stat 0.98  On top clinical + subendo |
|  | Cong et al. [12] 2014 ^†^ | **2D PLS** | + | ND | -10.8% | 90 | 92 | - | 2.56 [1.75- 3.85] | WMSI, LVEF, LAD, DT, ’ LVEDV, LVESV, troponin level WMSI PLS RS | - | X²= 22.8 |
|  |  | **2D PRS** | + | ND | 28.4 | 82 | 67 | - | 1.07 (1.02–1.13) |  |  | X²= 6.6 |
| *LV: left ventricle; EF ejection fraction; ESV: end systolic volume; EDV: end diastolic volume WMSI: wall motion score index, ANT: Anterior; CMR: cardiac magnetic resonance; CKMB: creatine phosphokinase MB; Trop: troponin T; Mo: Months, y: years, d: days. GE: General Electric; CV: cardiovascular; HF: Heart failure; STE: Speckle tracking echocardiography; 2D/3D PLS: Two/Three dimensional peak longitudinal strain; 2DPCS: 2D peak circumferential strain; 2DPRS: 2D peak radial strain; 2Dprin: 2D principal strain; Ap: apical; Bas: Basal; Rot: Rotation; subepi: subepicardial; subend: subendocardial; PTS: Peak transverse strain; FU: follow-up; STEMI: ST elevation myocardial infarction; NSTEMI: non ST* | | | | | | | | | | | | |

**Table D:** Association of deformation values with cardiac reverse LV remodeling

|  | **First author**  **(Ref. #),**  **Year** | **STE PARAMETER** | | **Association** | **AUC**  **(CI 95%)** | | **Determinant Cut-off value** | **Sensitivity, Specificity (%)** | | **UNIVARIATE ASSOCIATION**  **OR or B** | **MULTIVARIATE ASSOCIATION**  **OR or B** | **Candidate variables in the multivariable analysis** | **Other significant variables in the multivariable analysis** | **Predictive values**  **(C-Index- NRI or Chi2)** |
| --- | --- | --- | --- | --- | --- | --- | --- | --- | --- | --- | --- | --- | --- | --- |
| **REVERSE REMODELING** | Atiok et al [4] 2014 | **2DPLS** | | + | 0.71 (0.55-0.87) | | -12.9% | 72 | 69 | - | - | - | - | - |
|  | Mollema et al [13] 2010 ^*^ | **2DPLS** | | + | 0.87 (0.82-0.93) | | -13.7% | 86 | 74 | 0.46 [0.36-0.59] | 0.5 [0.38-0.68] | AFI global LV strain,  troponin T, CPK, WMSI,  LAD, use of diuretics, ARB | CPK | - |
|  | Spinelli et al [14] 2013 ^*^ | **2DPLS** | | - | ND | |  | - | - | 0.93 [0.82-1.06] | ‡ | Peak troponin EDVi LVEF, LV conic index, WMSI, E/A ratio, E/E’, pLS, LV twist, LV torsion | LV conic index- | - |
|  |  | **Twist** | | + | ND | |  | - | - | 2.8 [1.4-4.5] | ‡ |  |  |  |
|  |  | **Torsion** | | + | + | | 1.34° | 88 | 80 | 2.9 [2.3-4.1] | 2.3 [1.5-3.2] |  |  |  |
|  | Carasso et al [15] 2013 | **2D PLS** | | + | | 0.60 (0.48-0.71) | -10% | 59 | 59 | - | - | - | - | - |
|  |  | **2D PCS** | | + | 0.67 (0.55-0.79) | | -15% | 65 | 65 |  |  |  |  |  |
|  |  | **2D PRS** | | - | ND | | NS | NS | NS |  |  |  |  |  |
|  |  | **2D prin S** | | + | 0.66 (0.53-0.78) | | -19% | 68 | 68 |  |  |  |  |  |
|  | Park et al [16] 2012 ^*^ | **2DPLS** | anterior | + | - | | - | - |  | 0.77 [0.56-1.05] ^*^ 0.97 [0.72-1.32] ^*^ | ‡ | CK, LVESI, WMSI, E/E’, 2DPLS , LV twist LV torsion | none | - |
|  |  |  | inferior |  |  |  |  |  |  |  |  |  |  |  |
|  |  | **LV Torsion** | | + | 0.78 [0.61-0.90] | | 0.54°/cm | 50 | 100 | 0.14 [0.03-0.63] | 0.13 [0.02-0.75] |  |  |  |
|  |  | **LV twist** | |  |  | |  |  |  | 0.79 [0.65-0.95] | - |  |  |  |
|  | Abate et al [17] 2011 | **3D PLS** | | + | 0.96 | | -11.1% | 92 | 91 | B -0.640 | B -0.611 | LVE(D)SV, ant location, troponin, WMSI, 3DGLS | troponin | **Chi2** = 95.42  C stat 0.84 on top of clinical and TTE parameters |
|  | Szymczyk et al [18] 2014 | **2DLS** | | + | 0.77 | | -10.4% | - | - | - | - | - | - | - |
|  |  | **2DTS** | | - | - | | - | - | - |  |  |  |  |  |
|  | Park et al [19] 2010 | **2D PLS** | | - | NS | | - | - | - | - | - | LV EDV; ESV ; early diastolic mitral velocity deceleration time CFR CK-MB; A-strain | CPK | - |
|  |  | **Apical strain** | | + | ND | | -6.1% | 60 | 90 | - | 2.02 [1.03 -3.97] |  |  |  |
|  | Shehata et al [20] 2014 | **2DPLS** | | - | 0.5 (0.31-0.68) | | -9.5% | 50 | 50 | - | - | - | - | - |
|  | Bergerot et al [21] 2014 | **2DPLS** | | + | - | | - | - | - | B=-1.39 | ND | MI size, LAD, MVO | - | - |
|  | Korosoglou et al [22] 2008 | **2DPLS** | | + | 0.8 | | -10.1% | - | - | - | - | - | - | - |
|  | Orii et al [23] 2014 | **2D PLS** | | + | 0.72 (0.64-0.80) | | -12.6% | 72 | 62 | - | - | - | - | - |
|  |  | **2D PCS** | |  | 0.90 (0.85-0.95) | | -14.3% | 81 | 80 |  |  |  |  |  |
|  |  | **2D PRS** | |  | 0.68 (0.60-0.76) | | 23.1% | 63 | 72 |  |  |  |  |  |
| *LV: left ventricle; EF ejection fraction; ESV: end systolic volume; EDV: end diastolic volume WMSI: wall motion score index, ANT: Anterior; CMR: cardiac magnetic resonance; CKMB: creatine phosphokinase MB; Trop: troponin T; Mo: Months, y: years, d: days. GE: General Electric; CV: cardiovascular; HF: Heart failure; STE: Speckle tracking echocardiography; 2D/3D PLS: Two/Three dimensional peak longitudinal strain; 2DPCS: 2D peak circumferential strain; 2DPRS: 2D peak radial strain; 2Dprin: 2D principal strain; Ap: apical; Bas: Basal; Rot: Rotation; subepi: subepicardial; subend: subendocardial; PTS: Peak transverse strain; FU: follow-up; STEMI: ST elevation myocardial infarction; NSTEMI: non ST* | | | | | | | | | | | | | | |

*** univariate retained in the meta-analysis; † multivariate retained in the meta-analysis, ‡ not retained in the multivariable model .**

LV: left ventricle; EF ejection fraction; ESV: end systolic volume; EDV: end diastolic volume WMSI: wall motion score index, ANT: Anterior; CMR: cardiac magnetic resonance;

CKMB: creatine phosphokinase MB; Trop: troponin T; Mo: Months, y: years, d: days. GE: General Electric; CV: cardiovascular; HF: Heart failure;

STE: Speckle tracking echocardiography; 2D/3D PLS: Two/Three dimensional peak longitudinal strain; 2DPCS: 2D peak circumferential strain; 2DPRS: 2D peak radial strain; 2Dprin: 2D principal strain; Ap: apical; Bas: Basal; Rot: Rotation; subepi: subepicardial; subend: subendocardial; PTS**:** Peak transverse strain; FU: follow-up; STEMI: ST elevation myocardial infarction; NSTEMI: non ST elevation myocardial infarction; PTCA: Primary cutaneous coronary angioplasty; MVO : Micro vascular obsctruction

AUROC: area under the receiver operation curve; CI: confidence interval; OR: odds ratio; NRI: Net reclassification improvement index; C Stat, X²: Chi 2

NA: data were not available or that the category is not applicable.

**REFERENCES**

1. Joyce E, Hoogslag GE, Leong DP, Debonnaire P, Katsanos S, Boden H, et al. Association Between Left Ventricular Global Longitudinal Strain and Adverse Left Ventricular Dilatation After ST-Segment-Elevation Myocardial Infarction. Circ-Cardiovasc Imaging. 2014 Jan;7(1):74–81.

2. Park YH, Kang S-J, Song J-K, Lee EY, Song J-M, Kang D-H, et al. Prognostic value of longitudinal strain after primary reperfusion therapy in patients with anterior-wall acute myocardial infarction. J Am Soc Echocardiogr. 2008 Mar;21(3):262–7.

3. Bochenek T, Wita K, Tabor Z, Grabka M, Krzych Ł, Wróbel W, et al. Value of Speckle-Tracking Echocardiography for Prediction of Left Ventricular Remodeling in Patients with ST-Elevation Myocardial Infarction Treated by Primary Percutaneous Intervention. Journal of the American Society of Echocardiography. 2011 Dec;24(12):1342–8.

4. Altiok E, Tiemann S, Becker M, Koos R, Zwicker C, Schroeder J, et al. Myocardial Deformation Imaging by Two-Dimensional Speckle-Tracking Echocardiography for Prediction of Global and Segmental Functional Changes after Acute Myocardial Infarction: A Comparison with Late Gadolinium Enhancement Cardiac Magnetic Resonance. J Am Soc Echocardiogr. 2014 Mar;27(3):249–57.

5. Zaliaduonyte-Peksiene D, Vaskelyte JJ, Mizariene V, Jurkevicius R, Zaliunas R. Does Longitudinal Strain Predict Left Ventricular Remodeling after Myocardial Infarction?: Longitudinal Strain Predicts Left Ventricular Remodeling. Echocardiography. 2012 Apr;29(4):419–27.

6. Hung C-L, Verma A, Uno H, Shin S-H, Bourgoun M, Hassanein AH, et al. Longitudinal and Circumferential Strain Rate, Left Ventricular Remodeling, and Prognosis After Myocardial Infarction. Journal of the American College of Cardiology. 2010 Nov;56(22):1812–22.

7. Haberka M, Liszka J, Kozyra A, Finik M, Gąsior Z. Two-Dimensional Speckle Tracking Echocardiography Prognostic Parameters in Patients after Acute Myocardial Infarction. Echocardiography. 2014 Jun 28;

8. Bonios MJ, Kaladaridou A, Tasoulis A, Papadopoulou E, Pamboukas C, Ntalianis A, et al. Value of apical circumferential strain in the early post-myocardial infarction period for prediction of left ventricular remodeling. Hellenic J Cardiol. 2014 Aug;55(4):305–12.

9. Jang JY, Woo JS, Kim W-S, Ha SJ, Sohn IS, Kim W, et al. Serial Assessment of Left Ventricular Remodeling by Measurement of Left Ventricular Torsion Using Speckle Tracking Echocardiography in Patients With Acute Myocardial Infarction. Am J Cardiol. 2010 Oct 1;106(7):917–23.

10. Nucifora G, Marsan NA, Bertini M, Delgado V, Siebelink H-MJ, van Werkhoven JM, et al. Reduced left ventricular torsion early after myocardial infarction is related to left ventricular remodeling. Circ Cardiovasc Imaging. 2010 Jul;3(4):433–42.

11. Abate E, Hoogslag GE, Leong DP, Bertini M, Antoni ML, Nucifora G, et al. Association between multilayer left ventricular rotational mechanics and the development of left ventricular remodeling after acute myocardial infarction. J Am Soc Echocardiogr. 2014 Mar;27(3):239–48.

12. Cong T, Sun Y, Shang Z, Wang K, Su D, Zhong L, et al. Prognostic Value of Speckle Tracking Echocardiography in Patients with ST-Elevation Myocardial Infarction Treated with Late Percutaneous Intervention. Echocardiography. 2014 Dec 4;

13. Mollema SA, Delgado V, Bertini M, Antoni ML, Boersma E, Holman ER, et al. Viability assessment with global left ventricular longitudinal strain predicts recovery of left ventricular function after acute myocardial infarction. Circ Cardiovasc Imaging. 2010 Jan;3(1):15–23.

14. Spinelli L, Morisco C, Assante di Panzillo E, Izzo R, Trimarco B. Reverse left ventricular remodeling after acute myocardial infarction: the prognostic impact of left ventricular global torsion. Int J Cardiovasc Imaging. 2013 Apr;29(4):787–95.

15. Carasso S, Agmon Y, Roguin A, Keidar Z, Israel O, Hammerman H, et al. Left ventricular function and functional recovery early and late after myocardial infarction: a prospective pilot study comparing two-dimensional strain, conventional echocardiography, and radionuclide myocardial perfusion imaging. J Am Soc Echocardiogr. 2013 Nov;26(11):1235–44.

16. Park S-M, Hong S-J, Ahn C-M, Kim Y-H, Kim J-S, Park J-H, et al. Different impacts of acute myocardial infarction on left ventricular apical and basal rotation. Eur Heart J Cardiovasc Imaging. 2012 Jun;13(6):483–9.

17. Abate E, Hoogslag GE, Antoni ML, Nucifora G, Delgado V, Holman ER, et al. Value of Three-Dimensional Speckle-Tracking Longitudinal Strain for Predicting Improvement of Left Ventricular Function After Acute Myocardial Infarction. Am J Cardiol. 2012 Oct 1;110(7):961–7.

18. Szymczyk E, Lipiec P, Michalski B, Szymczyk K, Shim A, Woźniakowski B, et al. 2D speckle tracking echocardiography for the assessment of regional contractile reserve after myocardial infarction. J Cardiovasc Med (Hagerstown). 2014 Oct 9;

19. Park S-M, Hong S-J, Kim Y-H, Ahn C-M, Lim D-S, Shim W-J. Predicting myocardial functional recovery after acute myocardial infarction: relationship between myocardial strain and coronary flow reserve. Korean Circ J. 2010 Dec;40(12):639–44.

20. Shehata M. Value of Two-Dimensional Strain Imaging in Prediction of Myocardial Function Recovery after Percutaneous Revascularization of Infarct-Related Artery. Echocardiography. 2014 Nov 24;

21. Bergerot C, Mewton N, Lacote-Roiron C, Ernande L, Ovize M, Croisille P, et al. Influence of microvascular obstruction on regional myocardial deformation in the acute phase of myocardial infarction: a speckle-tracking echocardiography study. J Am Soc Echocardiogr. 2014 Jan;27(1):93–100.

22. Korosoglou G, Haars A, Humpert PM, Hardt S, Bekeredjian R, Giannitsis E, et al. Evaluation of myocardial perfusion and deformation in patients with acute myocardial infarction treated with primary angioplasty and stent placement. Coron Artery Dis. 2008 Nov;19(7):497–506.

23. Orii M, Hirata K, Tanimoto T, Shiono Y, Shimamura K, Ishibashi K, et al. Two-Dimensional Speckle Tracking Echocardiography for the Prediction of Reversible Myocardial Dysfunction after Acute Myocardial Infarction: Comparison with Magnetic Resonance Imaging. Echocardiography. 2014 Aug 11;
